# The geometry of photopolymerized topography influences neurite pathfinding by directing growth cone morphology and migration

**DOI:** 10.1088/1741-2552/ad38dc

**Published:** 2024-04-04

**Authors:** Joseph T Vecchi, Madeline Rhomberg, C Allan Guymon, Marlan R Hansen

**Affiliations:** 1 Department of Molecular Physiology and Biophysics, University of Iowa, Iowa City, IA, United States of America; 2 Department of Otolaryngology Head-Neck Surgery, University of Iowa, Iowa City, IA, United States of America; 3 Department of Chemical and Biochemical Engineering, University of Iowa, Iowa City, IA, United States of America

**Keywords:** spiral ganglion neuron, neurite guidance, growth cone, pathfinding, dorsal root ganglion neuron, photopolymerization

## Abstract

*Objective*. Cochlear implants provide auditory perception to those with severe to profound sensorineural hearing loss: however, the quality of sound perceived by users does not approximate natural hearing. This limitation is due in part to the large physical gap between the stimulating electrodes and their target neurons. Therefore, directing the controlled outgrowth of processes from spiral ganglion neurons (SGNs) into close proximity to the electrode array could provide significantly increased hearing function. *Approach.* For this objective to be properly designed and implemented, the ability and limits of SGN neurites to be guided must first be determined. In this work, we engineer precise topographical microfeatures with angle turn challenges of various geometries to study SGN pathfinding and use live imaging to better understand how neurite growth is guided by these cues. *Main Results.* We find that the geometry of the angled microfeatures determines the ability of neurites to navigate the angled microfeature turns. SGN neurite pathfinding fidelity is increased by 20%–70% through minor increases in microfeature amplitude (depth) and by 25% if the angle of the patterned turn is made obtuse. Further, we see that dorsal root ganglion neuron growth cones change their morphology and migration to become more elongated within microfeatures. Our observations also indicate complexities in studying neurite turning. First, as the growth cone pathfinds in response to the various cues, the associated neurite often reorients across the angle topographical microfeatures. Additionally, neurite branching is observed in response to topographical guidance cues, most frequently when turning decisions are most uncertain. *Significance.* Overall, the multi-angle channel micropatterned substrate is a versatile and efficient system to assess neurite turning and pathfinding in response to topographical cues. These findings represent fundamental principles of neurite pathfinding that will be essential to consider for the design of 3D systems aiming to guide neurite growth *in vivo*.

**Abbreviations**
BDNF: Brain-derived neurotrophic factor
CI: Cochlear implant
DRGN: Dorsal root ganglion neuron
FBS: Fetal bovine serum
HBSS: Hanks’ balanced salt solution
HDDMA: 1,6-hexanediol dimethacrylate
HMA: hexyl methacrylate
NF200: High molecular weight neurofilament protein
NT-3: Neurotrophin-3
PBS: Phosphate buffered saline
SEM: Scanning electron microscopy
SGN: Spiral ganglion neuron.

## 1. Introduction

CIs are prosthetics that replace the mechanosensory transduction of sound by directly stimulating SGNs to provide hearing sensation. Tremendous improvements have been made to neural electrode devices in recent years, but they fail to fully emulate the sensory functions they seek to restore [1–3]. In particular, CIs are limited by the number of independent perceivable channels they provide [4, 5]. Much of the unrealized potential of this device arises from poor tissue integration, with a large distance between the stimulating electrodes and their target, SGNs in the spiral ganglion [6–8]. CI electrode arrays are typically inserted in the scala tympani and reside hundreds of microns away from the SGN somata in the ganglion, compared to the tens of nanometer gap of the synapse it is seeking to functionally replace. Enhancing the neural—electrode interface by reducing this gap is an essential element of next generation neural prostheses, as researchers seek to recapitulate the architecture and function of native neural systems more accurately [9–12].

To improve the ability of neural prostheses to mimic native neural function, there is intense interest in developing technologies that could guide axon regeneration into close proximity to the stimulating electrode [11, 13]. Further, this aspiration is not limited to SGNs and CIs as others are working to direct the growth of other neurons, such as DRGNs [14, 15]. To inform these aims, further work is needed to understand the fundamental behaviors of growth cones as they sense and respond to cues in their environment. Thus, many are studying how neurites grow and turn in response to various cues such as diffusible chemical gradients [10, 16], patterned peptide surface coatings [17, 18], and engineered surface topography [19–21], among others [22], including electrospun scaffolds [23, 24]. Though each of these approaches has its optimal uses and limitations, engineered surface topography to direct cell material interactions is particularly appealing for directing neurite growth to improve neural prostheses.

First, topographical cues offer tremendous control of cell behavior with precise modifications and reproducibility [25]. These engineered features can be controlled with submicron resolution and the pattern geometry (periodicity, amplitude/depth, and shape) can be easily modulated to explore the relationship between the topography and neurite guidance [19]. Additionally, methacrylate polymer systems are shelf stable and are used extensively in a variety of biomedical implant settings [26, 27]. Third, topographical growth cues are biologically relevant. Tissues present relevant micro-topographies that growth cones must navigate. Both topographical and biochemical cues activate similar signaling to direct growth cone guidance behavior [28, 29]. Finally, gradual, micron-scale, topographical cues can be engineered to offer similar or even greater influence on neurite guidance compared to biochemical cues [17, 18].

Prior work has indicated that biophysical cues dictate neurite guidance and behavior, however, the studies generally use simple geometries, repeating cues, or electospun fibers [30–32]. A significant, remaining question in neurite guidance by microtopographical features is what are the characteristics, abilities, and limits of a regenerating sensory neurite when being guided to turn in response to topographical cues [33]. Neurite turning has been studied extensively, however, most commonly using diffusible biochemical cues, which lack the precision and reproducibility of engineered biophysical cues [34, 35]. Thus, studies which systematically analyze the relationship of neurite turning to various topographies and turn geometries would provide valuable information for future translational applications that combine biomaterials with surface patterns to guide neurite growth [36, 37]. Subsequent regenerative growth will need to navigate through a complex milieu of matrix proteins and cells consisting diverse biophysical cues [38]. Therefore, greater work is needed to define how neurites navigate turning in response to biophysical cues, so that the characteristics and abilities of neurites to make these turns can be incorporated into designs for platforms intended for translation into *in vivo* environments.

By using the precision and reproducibility enabled by topographically engineered cues, we can accurately define neurite turning behavior in response to a range of angles, as well as various amplitudes of these angled microfeatures. This reproducible system is essential for studying neurite turning since neurite outgrowth and growth cone migration exhibit a wide variation of behavior across a population of neurons [39, 40]. Clarifying the consistency of how regenerating neurites can be effectively guided and determining what is the innate variation for this behavior are essential for informing the design of neural growth [41]. In future translational applications, where guided neurite growth is induced, it will be necessary to know what proportion of neurites will be properly guided by the cues utilized [42]. Further work is needed to address the efficacy of neurite guidance to biophysical turn cues of complex geometries.

For this work, primary sensory neurons were grown on novel topographically engineered substrates which present features with six different angle turn challenges. The amplitude of these features was varied by altering the photopolymerization parameters. To study neurite pathfinding generally, we assess neurite growth of both SGNs and DRGNs in response to this topography. The simple morphology of SGNs allows for detailed description of growth morphology, while the more dynamic DRGNs enable dynamic analysis of growth cone behavior and live imaging. In this work, we leverage the precision of photopolymerized topographical substrates to study the fundamental behaviors of neurite turning in response to a range of topographical cues, thereby informing the limits of pathfinding during neurite regeneration.

## 2. Methods

### 2.1. Micropatterned substrates

**Figure 1.**
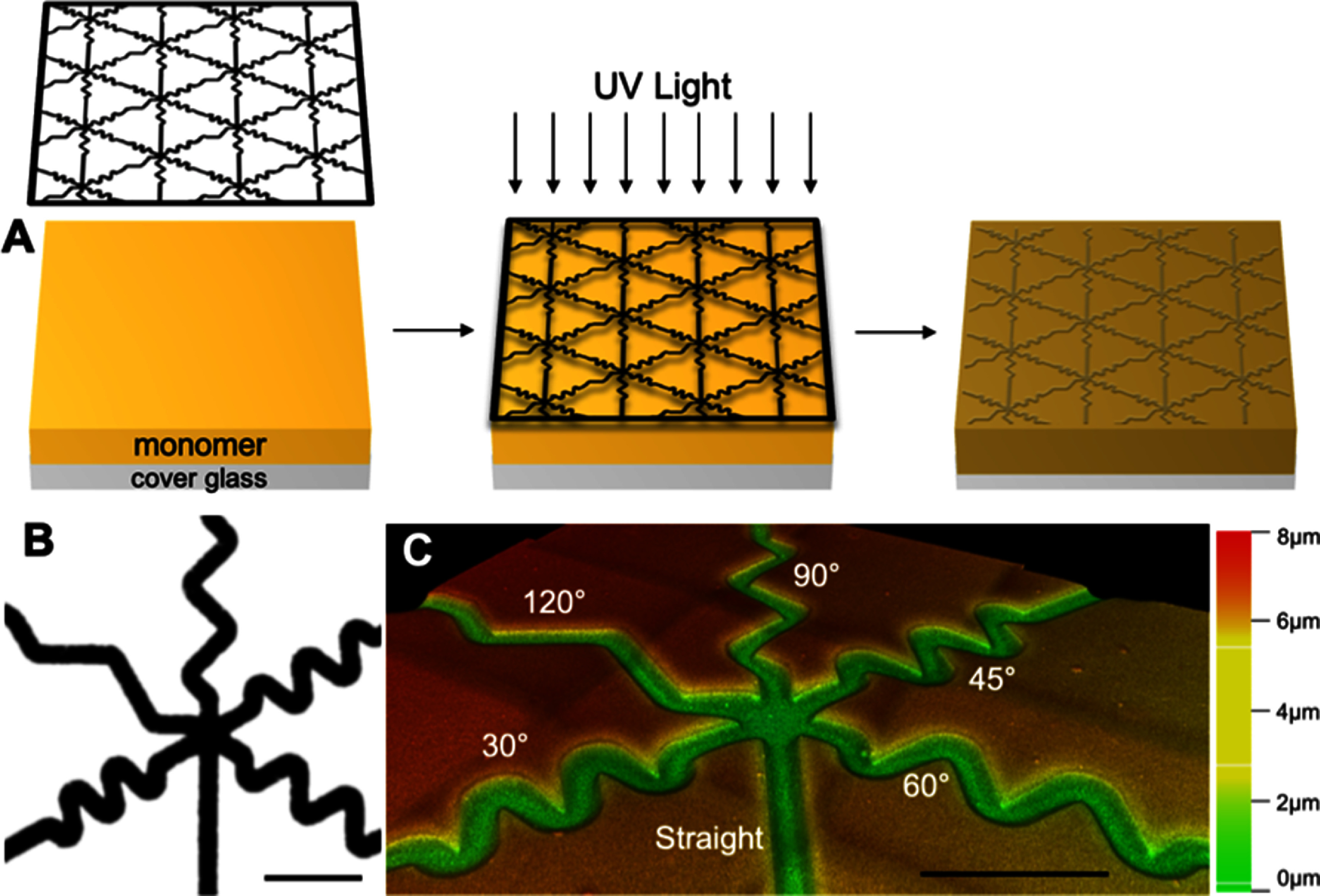
Schematic of photopatterning process and pattern characterization. (A) Schematic of photopolymerizing micropatterned substrates. A monomer solution (yellow) is added to a silane coupled glass cover slip (grey). Then the photomask (black outlined pattern) is placed on top of the solution before exposing the system to UV (ultraviolet, 355 nm) light. The monomer polymerizes to form a solid substrate and the photomask is removed. Schematic is representative and not to scale. (B) The photomask (black). This pattern repeats over the photomask to create the topographically micropatterned substrate. (C) A depth, color-coded confocal microscopy image of multi-angled microfeatures patterned in the substrate surface. Scale bars = 100 *μ*m.

Topographically micropatterned substrates were generated as previously described using photopolymerization (figure 1(a)) [19]. First, a monomer solution was formulated consisting of 40 wt% HMA (Aldrich), 59 wt% 1,6-hexanediol dimethacrylate (HDDMA, Aldrich), and 1 wt% of 2,2-dimethoxy-2-phenylacetophenone (DMPA, BASF). Then, to create the patterned substrates, this solution was evenly dispersed on a silane-coupled piece of cover glass by placing glass-chrome custom photomask (Applied Image Inc., figure 1(b)) on top. These samples were then exposed to 365 nm light at an intensity of 16 mW cm^−2^ using a high-pressure mercury vapor arc lamp (Omnicure S1500, Lumen Dynamics, Ontario, Canada) to polymerize the monomer solution (figure 1(a)). The opaque portions of the mask direct exposure of the UV radiation thereby modulating the rate of the polymerization locally to generate features on the surface. This process creates raised features or ridges underneath transparent bands where UV light intensity and the polymerization rate are highest. These substrates direct the growth of various cells and neurons, including SGNs and DRGNs [43]. Additionally, various geometries of topographical substrates can be generated by varying the photomask [34] or time of UV light exposure to change feature amplitude [19]. The duration of UV light exposure was changed in this work to create three microfeature amplitudes/depths of 2 *μ*m, 4 *μ*m, and 8 *μ*m with the custom photomask.

To generate multiangle features and micropatterns, a novel photomask was used to generate the micropatterns utilized here (figure 1). This custom photomask consists of 6 different microfeatures, i.e. channels with gradually sloped edges, that are 20 *μ*m in width arranged in a repeating hexagonal pattern. In addition to the control (straight) microchannel, the other five microfeatures consist of turns with varied angles (30°, 45°, 60°, 90°, and 120°) in which a neurite would need to deviate 20 *μ*m perpendicular to the trajectory of the microfeature to navigate the angled turn (labeled in blue on supplementary figure 1(a)). For the microfeature nomenclature, we defined the microfeature in reference to the interior angle with 30° being the sharpest turn, 120° the most gradual, and 45°, 60°, and 90° representing intermediate turn challenges.

Prior work suggests that neurites can bypass the angle turn challenges in situations with zero or small overlapping distance (distance perpendicular to the direction of the microchannel in blue on supplementary figure 1(a)) in the zigzags [34]. Therefore, we chose to design each angle turn challenge to have an equivalent distance (20 *μ*m) that the neurite would need to deviate perpendicular to the direction of the feature to navigate the turn. As such, the length of the straight portion of each microfeature’s zigzag is varied in order to standardize this design constraint (supplementary figure 1(c)).

### 2.2. Characterization of micropatterned substrate

The dimensions and characteristics of these micropatterned substrates were assessed using three methods. First, the topography of every substrate used in the following experiments was measured using white light interferometry (Dektak Wyko 1100 Optical Profiling System, Veeco; supplementary figure 2(a)). Feature amplitude was characterized in five regions of the glass coverslip and neurons were only cultured on areas within ±10% of the target microfeature amplitude. Vision software (Bruker) was used to create 3D images of substrates using this measurement technique.

A second method to characterize the micropatterned surface was SEM (Hitachi S-3400N; supplementary figure 2(b)). Conductive tape was used to mount sample substrates to SEM stubs and each sample was sputter coated with carbon prior to microscopy examination. The specimen was imaged top–down to assess the polymer surface.

The third method to assess the micropattern feature dimensions was confocal microscopy (STELLARIS 8, Leica; figure 1(c) and supplementary figure 2(c))). This approach was possible since the polymer interacts with ultraviolet light, therefore, enabling it to be imaged via confocal microscopy using a 405 nm laser. By creating a z-stack, the topographical dimensions as well as the character of the substrate surface can be determined.

### 2.3. Animals

All procedures involving animals were conducted in accordance with the NIH Guide for the Care and Use of Laboratory Animals and were approved by the University of Iowa Institutional Animal Care and Use Committee. All mice were maintained on a C57BL/6 (Envigo) background, housed in groups on a standard 12:12 h light: dark cycle with food and water provided ad libitum, and used at p3-5 of age when SGN peripheral processes already contact hair cells in the organ of Corti. For live cell imaging, neurons were harvested from Pirt-GCaMP3 transgenic mice [44].

### 2.4. SGN cultures

One day before culturing, substrates were sterilized by soaking in 70% ethanol for 2 min and then exposing them to UV light in a cell culture hood for 15 min. Cloning cylinders were placed on the patterned coverslip and then filled with poly-L-ornithine solution (Sigma-Aldrich) for 1 h at RT. After washing the substrate with sterile Milli-Q^®^ water, a laminin solution was added (20 *μ*g ml^−1^, Sigma-Aldrich) and incubated overnight at 4 °C. SGN cultures were prepared from mice between post-natal day 3 and 5 (p3-p5). Mice were decapitated, and their cochleae were isolated from their temporal bones in ice-cold PBS. The spiral ganglia were then isolated and placed into ice-cold HBSS(−/−) as previously described [45]. Enzymatic dissociation was then performed in calcium- and magnesium-free HBSS with 0.1% collagenase and 0.125% trypsin at 37 °C for 25 min. 100 *μ*l of FBS was added to stop dissociation. Ganglia were washed with Neurobasal media before being placed in supplemented Neurobasal Medium (Thermo Fisher) containing: 5% FBS, 2% N2 Supplement (Thermo Fisher), 10 *μ*g ml^−1^ Insulin, 50 ng ml^−1^ BDNF (R&D Systems), and 50 ng ml^−1^ NT-3 (Sigma-Aldrich). Once in supplemented media, ganglia were triturated first with a 1000 *μ*l pipette tip followed by a 200 *μ*l tip. Cultures were plated onto micropatterned substrates and maintained in a humidified incubator with 6% CO_2_ for 48 h.

### 2.5. Replated DRGN cultures

DRGNs were isolated as previously described [46], however, using neonatal (p3-p5) mice. First, a 24-well polystyrene plate was coated with poly-L-ornithine solution (Sigma-Aldrich) for 1 h at RT. The surface was washed with sterile Milli-Q^®^ water three times prior to a laminin solution (20 *μ*g ml^−1^, Sigma-Aldrich) being added and incubated overnight at 4 °C. After warming the coated well plate for 1 h at 37 °C, the freshly dissected DRGNs were cultured on this plate for 72 h before being replated. The replating procedure has been described previously [47], but after a 1 min incubation with TrypLE Express (Thermo Fisher), warm media was used to gently triturate the culture surface and lift the adhered neurons. The resulting replated DRGNs (rDRGNs) were then cultured on the micropatterned or unpatterned HDDMA/HMA substrates in a humidified incubator with 6% CO_2_ for 24 h.

### 2.6. Immunofluorescent labeling

After culture, media was removed and the culture surface was washed three times with phosphate-buffered saline without calcium and magnesium (PBS(−/−)). 4% paraformaldehyde in PBS(−/−) (Fisher Scientific) was added to fix the neurons. After 20 min at RT, cells were washed with PBS(−/−) three times, and then blocking buffer was added (5% normal goat serum [ThermoFisher], 0.2% Triton™ X-100 [Fisher Scientific], and 1% BSA [Research Products International] in PBS[−/−]). After incubating for 30 min at RT, different approaches were taken for the antibodies. For SGNs, mouse monoclonal anti-NF200 antibody (RRID: AB_260781, Millipore Sigma) was added (1:400 in blocking buffer) for 2 h at 37 °C. Following this incubation, the culture surface was washed 3 times with PBS (−/−) and then Goat anti-mouse Alexa Fluor^®^546 (RRID: AB_2534089, ThermoFisher) in blocking buffer (1:800) was added for 1 h at RT in the dark.

For the rDRGNs, chicken polyclonal antibody to NF200 (RRID: AB_2313552, Aves) was added (1:800 in blocking buffer) for 1 h at RT. Following this incubation, the culture surface was washed three times with PBS (−/−) and then Goat anti-Chicken Alexa Fluor^®^546 (RRID: AB_2534097, ThermoFisher) in blocking buffer (1:1000) was added for 1 h at RT in the dark. Following the secondary antibody incubation, both systems were treated the same; cultures were again washed with PBS (−/−) three times. Lastly, the polymer-coated cover glass was mounted onto a glass slide using Fluoromount-G^®^ (SouthernBiotech) and kept in the dark for 24 h before imaging.

### 2.7. Semi-automated measurement of neurite length within microfeature

Every SGN neurite that encountered a micropatterned, topographical microfeature was assessed in this analysis, in which neurite length in the microfeature was measured using NeuronJ [48]. Neurite length in microfeature was measured from where the neurite entered the microfeature to where it either exited the microfeature or the endpoint of the neurite was reached. These length data were then grouped both by turn angle and microfeature amplitude. For this approach, the unpatterned control condition was generated by overlaying the shape of the 60° microfeature over a flat portion of the substrate, and length data was measured in the same manner as neurites that encountered this pseudo-pattern.

### 2.8. Manual assessment of neurite pathfinding

Another method for assessing neurite pathfinding behavior was qualitatively scoring every neurite encounter with a microfeature. Neurites were scored as either turning or not turning. In particular, two conditions for which they were scored as not turning were: (1) did not follow the microfeature; neurite growth is not oriented in the direction of the pattern and the neurite crosses out of the microfeature nearly perpendicular to the microfeature’s direction, and (2) the neurite follows the straight portion of the microfeature but fails to complete the turn challenge and leaves the microfeature. Two categories of neurites were deemed to have successfully turned in response to the microfeatures: (1) the neurite follows the microfeature and navigates one turn; the neurite enters the microfeature more than 30 *μ*m before the turn challenge and remains positioned in the microfeature 30 *μ*m past the vertex of the turning challenge, or (2) the neurite navigates multiple turns and/or the shaft is reoriented where it is aligned across multiple of the zigzagging microfeatures. Like the previous metric, these data were grouped both by turn angle and microfeature amplitude.

### 2.9. Live DRGN imaging

Neurons growing in response to these turns were imaged live. To do this, rDRGNs were cultured from mice expressing Pirt-GCaMP3, a genetically encoded calcium indicator to allow imaging with a standard GFP/FITC/488 filter. These neurons were cultured on substrates with 4 *μ*m and 8 *μ*m amplitude microfeatures for 18 h. At that timepoint, neurites that were currently growing in a microfeature and actively engaging with the ridge of the feature were selected randomly and imaged every 15 s for 1.5 h to capture the growth dynamics. These videos were assessed by tracking the growth cones that were in the microfeature at the beginning of the recording and determining if they remained in the microfeature throughout the 1.5 h of imaging.

### 2.10. Comparing rDRGN growth cone morphology on micropatterned substrate

To study the morphology and behavior of neuron growth cones in response to topographical growth cues, replated DRGNs (rDRGNs) were cultured on a different topographically patterned substrate consisting of repeating rows of ridges and grooves (10 *μ*m periodicity and 3 *μ*m amplitude). These micropatterns offered a consistent pattern and thus a systematic methodology for this analysis. The substrates were made in the same manner as described previously [19].

For this assessment, rDRGNs were cultured on these micropatterns and on unpatterned substrates, then immunofluorescently labeled as above; however, the secondary antibody step was modified to assess growth cone morphology. For the modified secondary antibody step, Goat anti-chicken Alexa Fluor^®^488 (RRID: AB_2534096, ThermoFisher) (1:800) and Alexa Fluor™ 546 Phalloidin (ThermoFisher) (1:200) in blocking buffer was added for 1 h at RT. Fixed growth cones were imaged via confocal microscopy (STELLARIS 8, Leica). Images of the growth cones were analyzed by two approaches using Imaris software. First, the prolate ellipticity of the shape of the growth cone was computed (supplementary equation (1)). For this analysis, the software approximates the shape of the growth cone as an ellipsoid and derives the prolate ellipticity, which is a measure for how elongated the growth cone is. This shape was assessed since others have shown that the shape of the growth cone is influenced by substrate cues, thus the micropatterned substrate here may cause the shape to become elongated [49, 50]. Second, the 2D shape of the growth cone was approximated as an ellipse and its major axis was derived. The angle made between this major axis and the neurite shaft was measured (figure 5).

### 2.11. Assessing SGN ability to remain in the microfeature around a turn

Since the neurons were encountering turn challenges of various microfeature amplitudes, the tendency of these neurons to keep their shaft in the microfeature around a turn was assessed. All the neurites that were deemed to have turned in the analysis above (section 2.8) were further studied and the proportion of these neurites that remained in the microfeature around the turn was determined.

For each microfeature turn angle, a mathematical definition of what characterizes a neurite holding its position around a turn was derived. For this definition, the greatest angle path a neurite could take from halfway down each straight segment and remain in the microfeatures was measured (supplementary figure 1(b)). This was found to be 134°, 138°, 142°, 151°, and 160° for the 30°, 45°, 60°, 90°, and 120° microfeature turn angles, respectively. ImageJ was used to measure the angle of the neurite’s shaft from the point on the shaft at the microfeature turn’s vertex to points on the shaft 30 *μ*m on each side of the vertex (supplementary figure 1(d)). Neurites with angle of tension lower than that of the theoretical path of least tension for its given microfeature angle were characterized as holding tension, while those with angles greater were categorized as not holding tension. Neurite shafts that did not remain in the microfeature at the point of the vertex of the microfeature turn were also categorized as not holding tension. These data were grouped by microfeature amplitude.

In order to better visualize the neurites that held tension and/or reoriented, confocal microscopy was used to confirm the observations made by epifluorescence image analysis. The images were color coded by *z*-position to determine what plane the neurite shafts were positioned in these angled microfeatures.

### 2.12. Analysis of SGN neurite branching

Neurite branching was also assessed as a function of the angle at which the neurite encountered the edge of the microfeature. First, the angle at which the neurite encountered the ridge was measured using the angle tool in ImageJ (supplementary figures 3(a) and (b)). Then each neurite was scored as either follow, exit, or branch (if it branches at the microfeature boundary wherein one branch follows and the other exits). For this approach, only the distal-most neurite encounter with the micropattern for each neurite was assessed. Additionally, as part of this approach, the proportion of neurites that successfully turned was also measured as a function of the angle they approached the microfeature ridge and microfeature amplitude.

## 3. Results

### 3.1. Generation and characterization of topographically engineered substrate with multi-angled microfeatures

Using a customized photomask designed with varied angles (30°–120°), photopolymerization techniques were used to generate methacrylate surfaces with the specified micropatterning consisting of grooves in masked regions (figure 1). By altering the duration of light exposure, patterns were engineered with microfeatures of varied amplitude, or depth (2 *μ*m, 4 *μ*m, and 8 *μ*m). Thus, the photomask dictates pattern geometry while light exposure determines microfeature amplitude. The micropatterned substrates were characterized using multiple methods. The first method is via white light interferometry (supplementary figure 2(a)). This method enabled measurement of microfeature amplitude quickly and accurately. The substrates were also imaged with SEM to confirm that the microfeature ridges consist of sloping transitions rather than the typical, abrupt on/off features characteristic of other engineered micropatterns from lithography, etching, etc. (supplementary figure 2(b)). Lastly, z-stacks created with confocal microscopy also allowed for the measurement of the amplitude of the microfeatures, visualization of the smooth nature of the surface, and additionally the creation of depth color-coded images to aid in the quantitative and qualitative assessment of the substrate (figure 1(c) and supplementary figure 2(c)).

### 3.2. Microfeature geometry determines the fidelity at which SGN neurites navigate microfeatures

**Figure 2.**
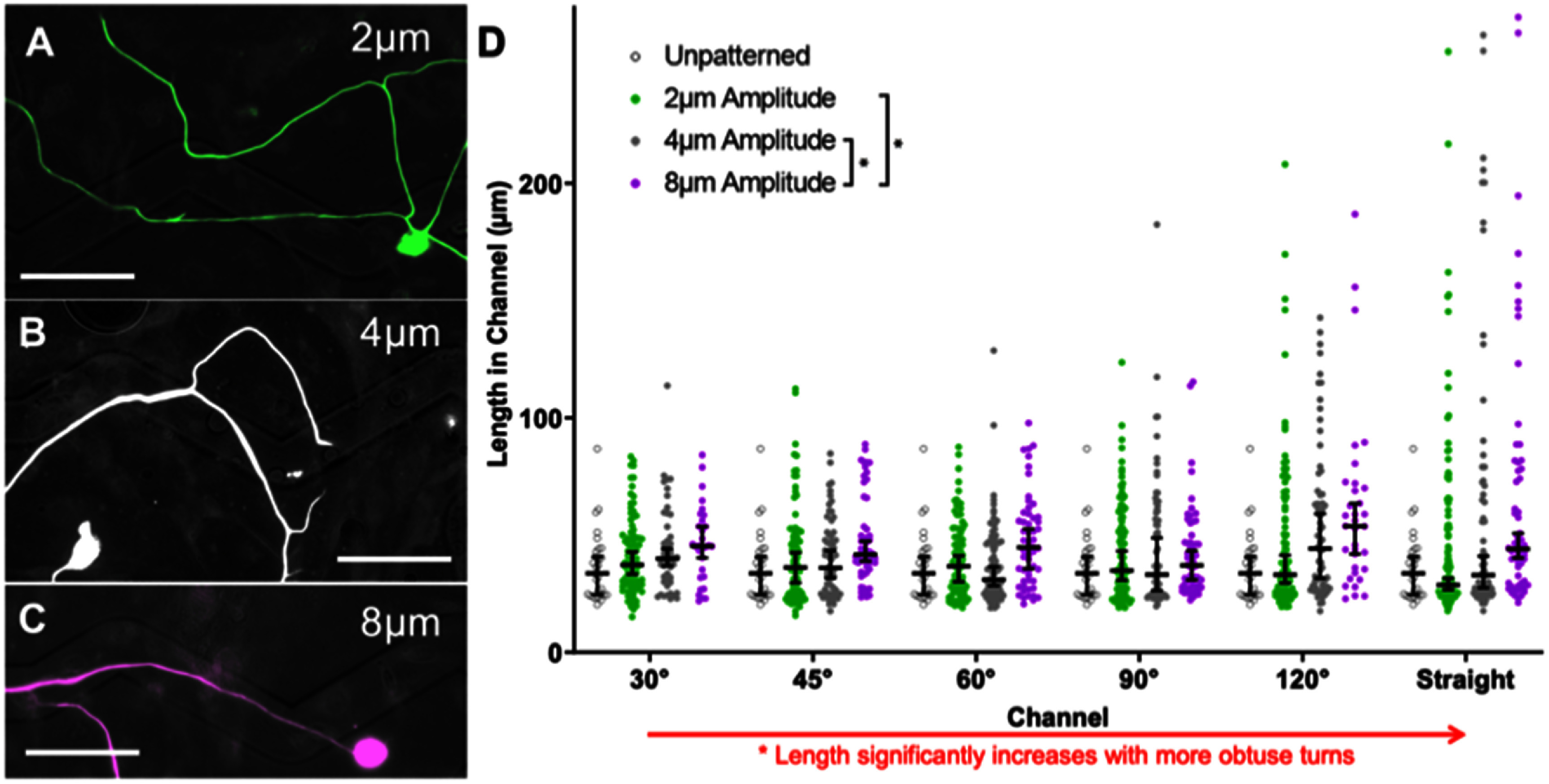
Feature geometry determines the ability of SGNs to navigate complex angle microfeature cues. (A)–(C). Representative epifluorescence images of spiral ganglion neuron (SGN) neurites encountering a 120° angle microfeatures across the amplitude conditions (A) 2 *μ*m (green), (B) 4 *μ*m (white), and (C) 8 *μ*m (purple). The SGNs were labeled with anti-NF200 antibodies. D. Scatter plots of the distance that SGN neurites follow a microfeature once encountering that feature. Median ±95% CI shown in black. n for each sub-condition ranges from 30 to 123 neurons traced. Unpatterned represents the shape of an angled microfeature superimposed onto a flat substrate. Since the data are not normally distributed and there are two sets of independent variables (channel and amplitude), a two-way ANOVA on ranks was conducted. This analysis shows that both microfeature amplitude and angle of turn affect the length that SGN neurites follow the microfeatures, with length increasing with deeper microfeatures and more obtuse turns. *p* < 0.01. Scale bars = 50 *μ*m.

The topographical multi-angled micropatterns enable the evaluation of neurite pathfinding in response to various turn challenges of precisely controlled geometry. In particular, SGN neurite pathfinding in response to six different microfeature turns (30°, 45°, 60°, 90°, 120°, and straight) was assessed across 3 different microfeature amplitudes (2 *μ*m, 4 *μ*m, and 8 *μ*m). Importantly, these are modest and biologically relevant growth cues consisting of gradually sloping transitions (figure 1(c) and supplementary figure 2). To provide an overall assessment of how strongly a neurite follows these various micropatterns, we measured the distance that each neurite remained in the microfeature channel and compared the data with two-way ANOVA. The analysis of neurite behavior on these substrates shows that the length of a neurite that remains in the microfeature increases both for more gradual turn challenges (straighter or larger angle microfeatures) as well as deeper microfeatures (figure 2). In particular, neurite length in a microfeature increases by an average of 20% from the 2 *μ*m (42.8 *μ*m) to 8 *μ*m amplitude (51.6 *μ*m). Thus, these results suggest that modest increases in channel depth enable neurites to be guided by the cues more efficiently. Additionally, the length also increases by an average of 23% from the sharpest turn to most gradual (42.7 *μ*m for 30° to 52.4 *μ*m for 120°). This implies that the neurites also exhibit greater fidelity in navigating more gradual turns.

**Figure 3.**
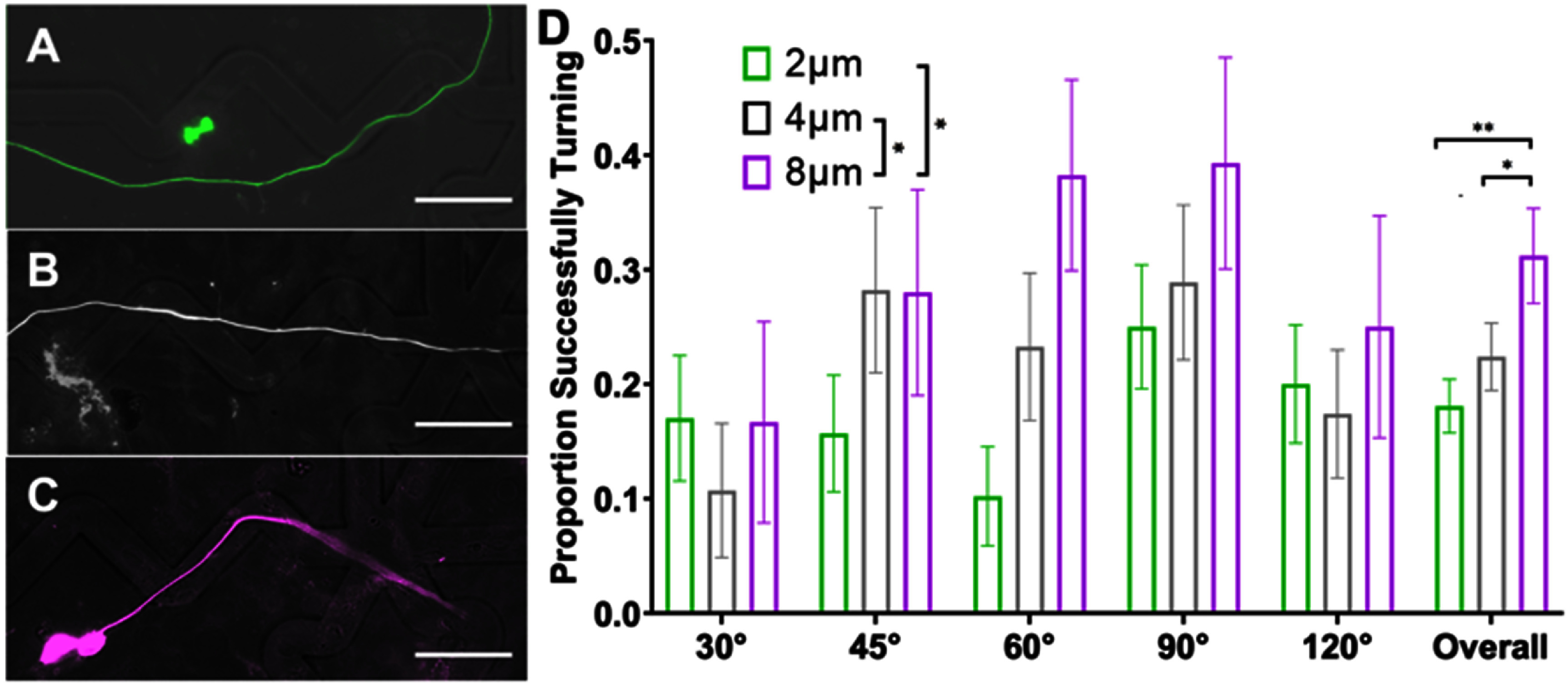
Microfeature amplitude promotes the ability of SGN neurites to turn. (A)–(C). Representative images of spiral ganglion neuron (SGN) neurites encountering a 90° angle microfeature across the amplitude conditions (A) 2 *μ*m (green) failing to turn, (B) 4 *μ*m (white) aligning across the microfeature, and (C) 8 *μ*m (purple) making a turn. The SGNs were labeled with anti-NF200 antibodies. (D) Proportion of SGN neurites that successfully navigate a given microfeature turn, *n* for each sub-condition ranges from 20 to 64 neurite encounters with a turn (supplementary table 1). Since the dependent variable is a proportion and there are two sets of independent variables (channel and amplitude), a multinomial logistic regression was used to assess the effect of channel geometry. This analysis shows that increasing microfeature amplitude improves the ability of SGN neurites to navigate turns. Since no effect was seen from turn angle, data were compiled by channel amplitude. The overall data were compared via Chi-square test with follow-up z-tests, indicating similarly that turning proportion increases with channel amplitude. Error bars represent ± standard error for a proportion for all data. *p* < 0.05. Scale bars = 50 *μ*m.

This conclusion is further evidenced by similar findings when the neurite behavior at each type of turn challenge is scored as noted in section 2.8. The proportion of neurites that successfully navigate the turns increases with microfeature amplitude increasing when assessed with multinomial logistic regression and chi-square (figure 3). On average, the percent of neurites that navigated the turns almost doubles from 18% making the turns on average in the 2 *μ*m amplitude microfeatures up to 31% in the 8 *μ*m amplitude microfeatures, a 72% increase in fidelity. In terms of microfeature angle, we see neurites are best able to turn in response to the moderate angle microfeatures (45°, 60°, 90°) (figure 3 and supplementary table 1). This second analysis does not perfectly isolate the variable of turn angle since the straight portion of the microfeature varies in length for each turn (supplementary figure 1(c)). Thus, the conclusions of the regression for this assessment are inconclusive and instead these data suggest that it is more challenging for a neurite to navigate a longer straight portion and more gradual turn (120° microfeature) than a shorter straight portion despite a sharper turn (45°, 60°, and 90° microfeatures). Lastly, we also see that regenerating neurites from another sensory neuron, rDRGNs, also successfully navigate these microfeatures in a geometry-dependent manner (supplementary figure 4).

### 3.3. Growth cones drive rDRGN neurite pathfinding

**Figure 4.**
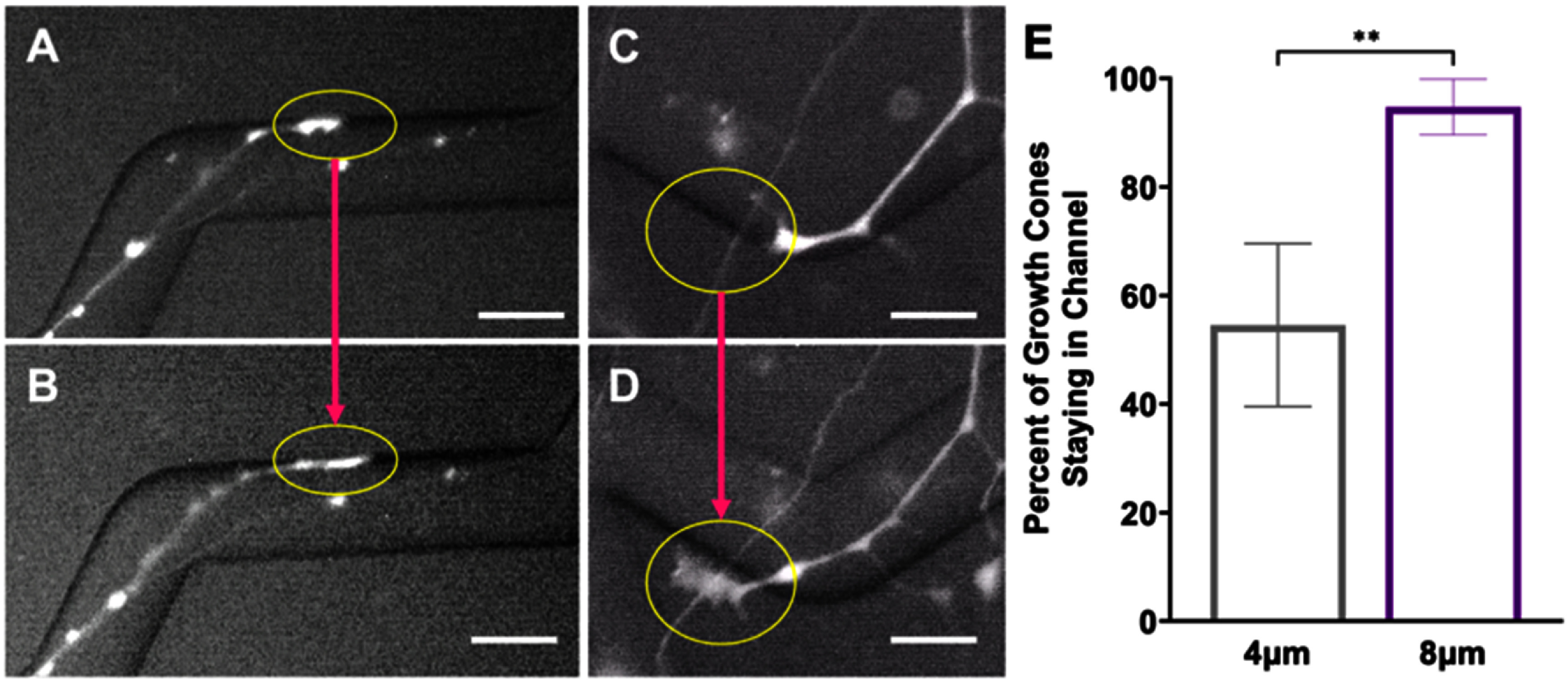
Growth cones are more likely to remain in deeper microfeatures. (A), (B) Representative epifluorescence images from supplemental video 1(a) in which a replated dorsal root ganglion neuron (rDRGN) growth cone encounters and remains in an 8 *μ*m amplitude, 120° angle microfeature through the course of 1.5 h of imaging. (C), (D) Representative epifluorescence images from supplemental video 1(b) in which a rDRGN growth cone encounters and exits a 4 *μ*m amplitude, 120° angle microfeature during 1.5 h of imaging. (E) Percent of rDRGN growth cones that remained in the microfeature throughout the 1.5 h recording. Data are proportions so a *Z*-test was conducted, which shows that a greater proportion of growth cones remain in the microfeature in the 8 *μ*m amplitude condition. *n* = 11 and 19 growth cones imaged. Error bars represent ± standard error for a proportion. *p* < 0.01. Scale bars = 20 *μ*m.

To better understand how neurites grow in response to these cues, replated DRGNs with a genetically encoded fluorescent indicator, Pirt-GCaMP3, were imaged live as they extended their neurites in the patterned angled microfeatures. In the videos, the growth cones were highly dynamic and strongly responded to the microfeature ridges in the 8 *μ*m amplitude condition (supplementary video 1). Based on the high fidelity with which the growth cones appeared to navigate the 8 *μ*m amplitude microfeatures, we compared the proportion of the growth cones that remained in the microfeature throughout the 1.5 h videos in the 4 *μ*m and 8 *μ*m amplitude microfeatures. Due to the low throughput of this analysis and the requirement that the growth cone of interest be actively engaging with a microfeature ridge at the beginning of recording, the growth cones analyzed were drawn from a random sample of turn angles and the growth cone positions within the microfeature (supplementary table 2). The proportion of growth cones that remained in the microfeature was much greater in the 8 *μ*m amplitude microfeatures (95% ± 5%) compared to the 4 *μ*m (55% ± 15%) (figure 4). In this analysis, we see the fidelity increase from about 50% guidance in the 4 *μ*m amplitude to nearly perfect guidance in the 8 *μ*m amplitude system. While the observed effect is robust, it is important to note that the duration of imaging (1.5 h) is shorter than the culture duration of the other studies (24 or 48 h).

### 3.4. rDRGN growth cones on micropatterned substrates have distinct morphology

**Figure 5.**
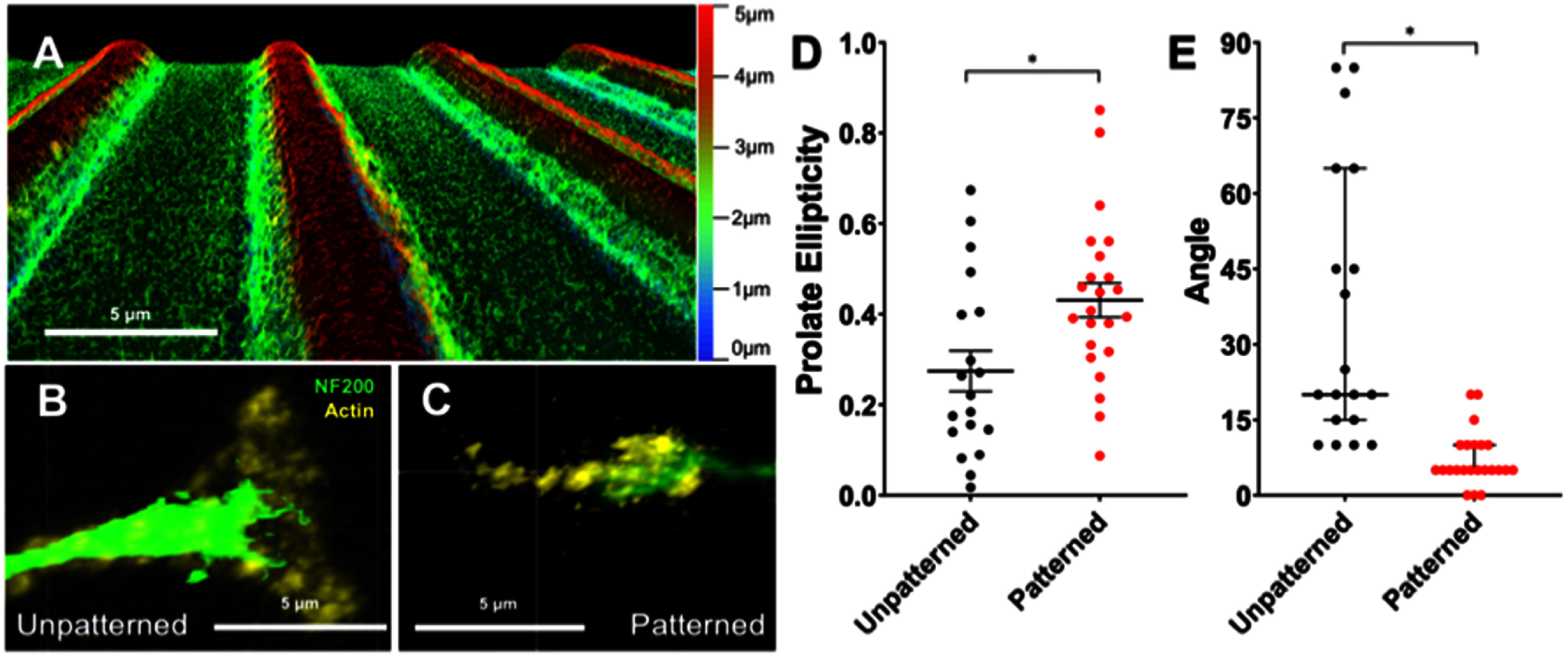
Growth cones exhibit distinct morphology and behavior on topographically micropatterned substrate. (A) Depth, color-coded confocal image of repeating rows of ridges and grooves substrate used for this experiment (3 *μ*m amplitude and 10 *μ*m periodicity). (B) Representative image of a replated dorsal root ganglion neuron (rDRGN) growth cone grown on an unpatterned substrate labeling with anti-NF200 antibodies and phalloidin (actin). (C) Representative image of rDRGN growth cone grown on the micropatterned substrate in (A). (D) rDRGN growth cone shape was approximated as a spheroid and its prolate ellipticity was calculated (supplementary equation (1)). Data are normally distributed and a t-test shows growth cones on the patterned substrate were more prolate. Error bars represent ±SEM, *p* < 0.05. (E) The major axis of this spheroid was found and the angle difference between the major axis and the neurite shaft was measured. Data are not normally distributed thus a Mann–Whitney shows that this angle was smaller for the neurons on the patterned substrate. Error bars represent 95% confidence interval. *p* < 0.001. *n* = 19 and 23 growth cones measured for both graphs. Scale bars = 5 *μ*m.

Given the stark difference in rDRGN growth cone pathfinding fidelity in different microfeature amplitudes in figure 4, we further assessed growth cone behavior on micropatterned substrates by growing rDRGNs on a topographical substrate consisting of narrow repeating rows of ridges and grooves (10 *μ*m periodicity and 3 *μ*m amplitude, figure 5(a)). We sought to determine if and how the growth cone morphology and orientation are altered when grown on topographically patterned substrates to inform how the strong differences in pathfinding in figure 4 manifests. When cultured on this micropatterned substrate, the ellipse shape of the rDRGN growth cones become more elongated and prolate than on unpatterned substrates, with prolate ellipticity values of 0.431 (±0.037) and 0.274 (±0.043), respectively (figure 5(d) and supplementary equation (1)). Additionally, the angle difference between the major axis of the growth cone and the neurite shaft was smaller in the neurons grown on the micropatterned substrate, a median difference of 5° compared to 20° on the unpatterned substrate (figure 5(e)). In combination with figure 4, these observations suggest both the importance of the growth cone in transducing the biophysical cues for proper pathfinding and that the growth cone can function as an assessment marker of pathfinding.

### 3.5. DRGN neurites reorient their shafts as they pathfind in response to microfeature turns, but not in a feature amplitude-dependent manner

After appreciating the significant role of the growth cone in figures 4 and 5, we next studied the behavior of the neurite shaft when growth cones are pathfinding in response to angled turn challenges. We observed that a significant subset of neurites aligned across, as opposed to with, the zig-zagged microfeatures in the fixed sample studies (figure 3(a), supplementary figure 5). To dynamically assess the behavior leading to this observation, live imaging of neurite shafts was conducted. The live imaging demonstrates that the neurite shafts are constantly reorienting their position as the neurite is elongating. The neurite shafts appear to be mobile and drift as the growth cone pathfinds (supplementary video 2). In particular, the neurites tend to reposition their shaft such that it becomes aligned in the direction of elongation. This movement thereby minimizes the number of neurites that hold their position in a zigzag or hold their tension around a turn. This behavior occurs since the neurite shaft moves to reposition itself outside of the microfeature and becomes more aligned in the direction of neurite elongation (supplementary video 2). Additionally, SEM images of these rDRGNs display neurites tightly following the microfeature ridge or just hopping over the vertex of the angle challenge of the microfeature (supplementary figure 5).

**Figure 6.**
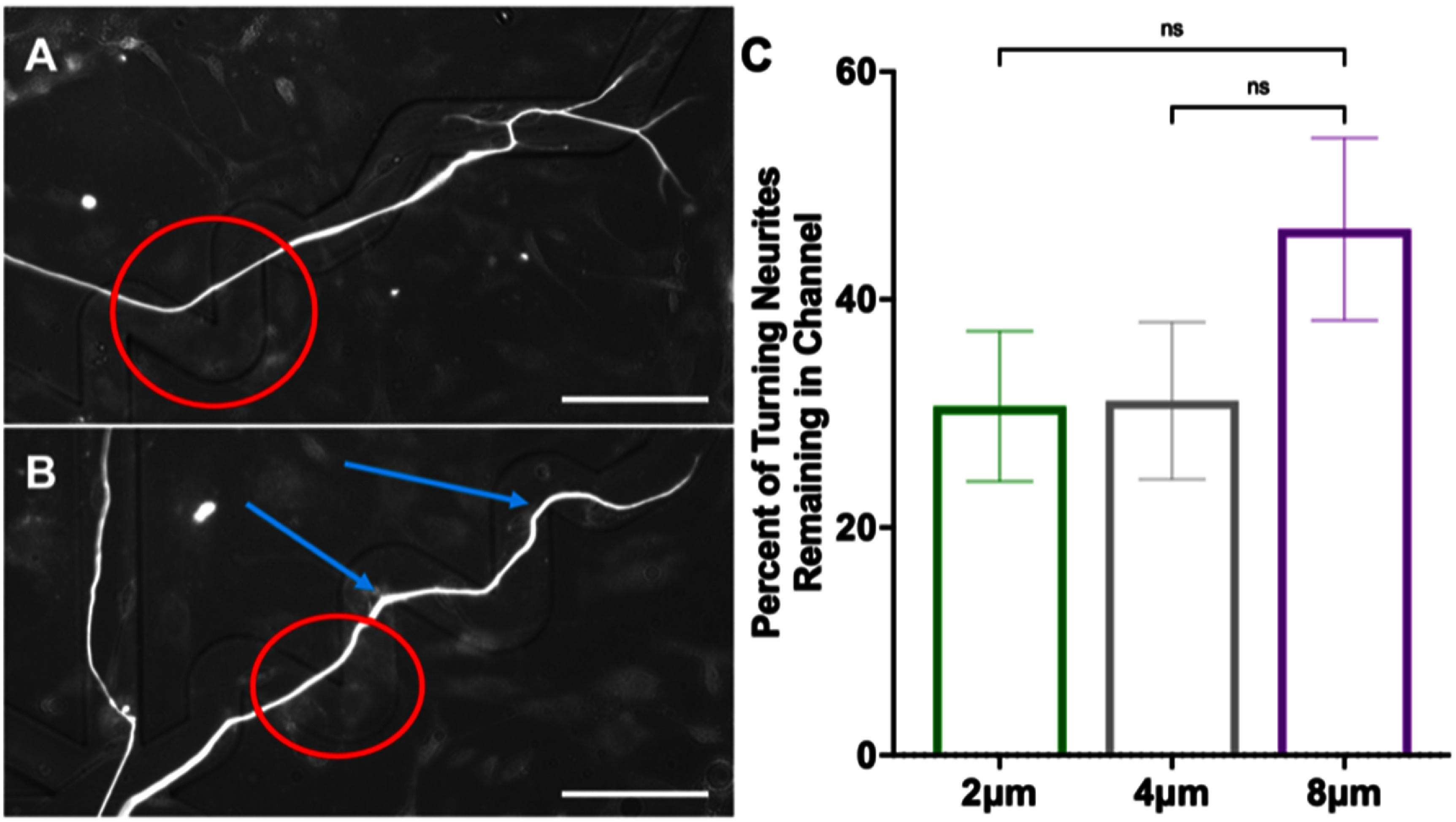
Neurite shafts remain in microfeatures at similar rates when navigating turns regardless of feature amplitude. (A) Representative epifluorescence image of a spiral ganglion neuron (SGN) neurite unable to hold position around a turn through a 4 *μ*m amplitude, 60° angle microfeature. (B) Representative image of SGN neurite holding position around two turns through an 8 *μ*m amplitude, 60° angle microfeature. Red circles indicate turns where the neurite shafts do not hold the turn while blue arrows show turns where neurites that do hold the turn. (C) The fraction of neurites that hold the position in the microfeature after successfully navigating a turn (supplementary table 3). Data are proportions from three treatment groups, thus a Chi square test was done which suggested no significant difference in treatment groups (*p* = 0.24). *n* = 49, 45, and 39 neurites observed to successfully make the microfeature turn. Error bars represent ± standard error for a proportion. Scale bars = 50 *μ*m.

The neurons that successfully turned in figure 3 were again assessed to determine if the proportion of neurites reorienting around the turns was related to microfeature geometry (supplementary table 3). In this analysis, there is no significant effect of feature amplitude on neurite shafts remaining in the microfeatures when making turns. Though a higher proportion of neurites remain in the microfeature when turning in the 8 *μ*m amplitude condition, this difference is not statistically significant via Chi square test since we are comparing a proportion in small subsets of the data and the magnitude of the difference is not large (31% vs. 46%, respectively) (figure 6). Additionally, no significant relationship was found between the angle of the turn and the ability of a neurite to hold tension when making turns. Despite the considerable reorientation exhibited by the neurites, this behavior appears to be a fundamental property of the neuron that is not affected by the modest changes to the microfeature amplitudes studied here.

### 3.6. Neurite branching is related to turning efficacy

During the live imaging of the neurite sharfs, the growth cones were observed to often branch when the growth cone encountered a ridge or turn challenges on the angled microfeatures. Additionally, the growth cones were observed to have a branched, arborizing appearance as they navigated the microfeatures (supplementary video 2). To quantitatively assess neurite branching, we scored neurite behavior (follow, exit, or branch) as a function of microfeature amplitude and the angle at which the neurite encountered a topographical ridge from within the angled microfeatures (supplementary figures 3(a) and (b)). This modified approach was used since the exact angle at which a growth cone encounters the feature ridge varies within each microfeature condition. Firstly, the results of this modified quantification echo the findings of figures 2 and 3 showing that the proportion of neurites successfully turning increases with greater microfeature amplitude and with more obtuse angles (supplementary figure 3(c)). The neurites are observed to better follow the microfeatures by a magnitude of at least 3.5-fold when they approach the microfeature ridge at a less severe angle. Additionally, neurites are seen to better follow as microfeature amplitude increases from 2 *μ*m to 8 *μ*m by an average of 2.8-times (supplementary figure 3(c)).

**Figure 7.**
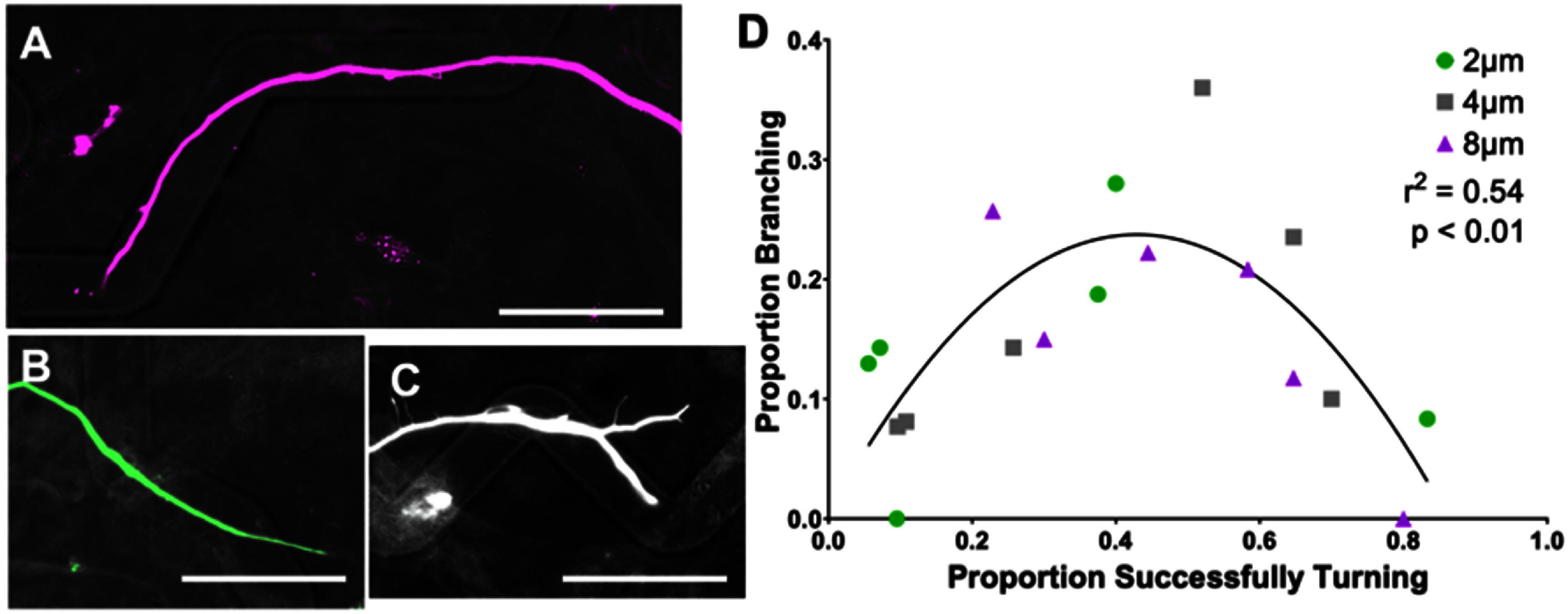
Neurite branching is most likely when neurite turning result is uncertain. (A) spiral ganglion neuron (SGN) neurite turning in response to an 8 *μ*m amplitude, 120° angle microfeature, representing an ‘easy turn’ toward the far right of the graph. (B) Epifluorescence image of SGN neurite exiting a 2 *μ*m amplitude microfeature represents a ‘challenging turn’ towards the left of the graph. (C) SGN neurite branching to both follow and exit the microfeature in a 4 *μ*m amplitude, 60° angle feature, representing an intermediate challenge where branching was seen to be most likely. (D) For each type of neurite encounter (angle of approach and channel amplitude), the proportion of neurites which successfully turns and that branched was determined. Here, we plotted neurite branching as a function of neurites successfully turning to assess their relationship. We sought to compare the relationship between these variables. Thus, a second order polynomial was generated to fit the data. We then compared how well the regression fit the data with an *F*-test and found *r* ^2^ = 0.54 which equates to *p* < 0.01. Scale bars = 50 *μ*m.

When studying the relationship between neurite turning and branching using this modified assessment, we see neurite branching occurs most frequently when the probability of neurite turning approximated 50% (figure 7). Branching is observed to be less likely to occur when the neurite turning outcome becomes more certain, i.e. when approaching no chance of turning or all neurites making the turn. The microfeature angle where branching was maximized varied across microfeature amplitudes. For the 2 *μ*m microfeatures, maximal branching occurs in gradual turns (15°–30°), 4 *μ*m in moderate turns (30°–45°), while sharper turn challenges maximized branching in the 8 *μ*m amplitude microfeature (75°–90°).

## 4. Discussion

Neural prostheses including CIs can restore lost function, however, the auditory perception and quality provided are significantly reduced compared to native pathways. CIs represent the most successful neural prostheses to date, but the large distance between the stimulating electrode and the target SGNs limits their function. Closing this physical gap to improve signal resolution is a primary goal with a variety of approaches proposed including; (1) altering the electrode array shape to place it in a perimodiolar position so that the stimulating electrodes reside closer to the neurons [51, 52], and (2) tissue engineering endeavors to induce de novo, organized neurite growth towards the stimulating electrodes [11, 53]. While CIs are our primary research interest, the conceptual findings of this study represent foundational work which translate broadly to neural regenerative approaches in all domains [38, 54]. For any endeavor seeking to engineer neurite guidance into an electrode interface, or any other material, design choices will need to be made for how to biochemically coat or biophysically pattern the material [10, 11, 13, 36]. Prior work has suggested that for optimal translational potential, shelf stability, biocompatibility, and precision of guidance should be prioritized [55]. In order to inform these design choices, foundational work is essential to describe the characteristics and abilities of neurites to be guided. This work here focuses on the role of topographical cues for neurite guidance. Thus, in this study we use the spatial and temporal control inherent to photopolymerization to create novel, precise topographically patterned substrates to guide neurite regeneration and thereby evaluate the ability of sensory neurons to navigate topographical angled turn challenges. Both SGNs and rDRGNs were assessed to study multiple sensory neurons and to use diverse neurons with different characteristics.

### 4.1. Growth cones respond to the topographically micropatterned substrate and drive neurite pathfinding and turning

Herein, we have demonstrated a novel system to assess the ability of neurites to turn in response to topographical cues of varied angles and amplitudes (depths). Using engineered micropatterned substrates, we see that the geometry of the angle turn challenge, i.e. microfeature amplitude and angle of turn, governs the ability of the sensory neurites to navigate the turn and follow the micropattern (figures 2 and 3). This conclusion appears straightforward; however, we see a wide variation in how neurites respond to the same geometric cue (figure 2). Furthermore, neurites can follow or not follow any microfeature studied implying, that in these patterned microfeatures, there is no distinct upper or lower limit of a neurite’s ability to navigate a turn (figure 3). Therefore, while the geometry of the angled features influences how well sensory neurites track the microfeature guidance cues (figure 2) and navigate the turns (figure 3), the process by which these neurites navigate these turns is not simply causal. These data suggest that as SGN neurites navigate complex turn challenges, their behavior reflects a balance of deterministic cues guiding their growth and their innate stochastic outgrowth patterns. Therefore, the strength of the guidance cue does not perfectly establish growth cone behavior, but rather shifts the probability distribution of growth cone turning behavior.

This random nature of growth cone motility has been described previously [56–58]. Neurite pathfinding results from an interplay between the strength of the turn cue [17, 19], stochastic actin polymerization [58], and signaling from the support cells from the primary culture influencing guidance cues [54]. Within the experimental system used here, these factors result in non-uniform pathfinding observations within each geometric condition, e.g. neurites do not navigate the gradual turns in the 8 *μ*m amplitude with perfect fidelity. It is important to note, that sufficiently strong cues can overpower the stochastic nature of the neurite pathfinding to create nearly deterministic systems [19, 59]. While the biophysical cues utilized here do not overpower the innate randomness, we show that these topographical cues, of biologically relevant dimensions, can strongly shift the probability distribution of neurite turning behavior. Thus, this is an appealing model to study turning and guidance since it reflects the balance of both directed and random behavior inherent to neurite guidance *in vivo* [60, 61]. Such a model of neurite turning in response to topography can be applied to study fundamental signaling mechanisms that contribute to neurite guidance and translation to *in vivo* applications.

To better understand the neurite pathfinding process, live imaging of rDRGN neurites navigating the microfeatures was conducted (supplementary video 1 and 2). In these videos, the growth cones showed a strong tendency to closely track the microfeature ridge for the 8 *μ*m amplitude microfeatures (95%), while in the shallower 4 *μ*m microfeature, the fidelity to the microfeature was less consistent (55%) (figure 4). These data suggest two main findings. First, since the growth cones consistently remain in deep microfeature (8 *μ*m amplitude), strong, biologically relevant topographical cues can be engineered that precisely direct neurite guidance and turning. Second, based on the degree of the difference observed in figure 4, it appears that growth cone behavior accounts for the differences in pathfinding that is observed in the prior analyses, figures 2, 3 and supplementary figure 4.

After observing differential rDRGN growth cone behavior to the various angled microfeature geometries, we further assessed how growth cone morphology and behavior change using a topographical substrate with narrow repeating rows of ridges and grooves. This system offered a systematic methodology and further support for growth cone behavior being involved in differential pathfinding to topographical cues. Importantly the growth cone is more prolate when grown in the microfeatures (figure 5(d)) as well as the growth cone appears elongated to become strongly oriented in the direction of the microfeatures and neurite shaft (figure 5(e)). The alignment of the growth cone and shaft suggests that changes in growth cone morphology and behavior underlie the pathfinding observations downstream. In particular, by integrating these findings with previous work, it appears that the topographically patterned substrate and growth cone interact to cause the growth cone to become elongated in the direction of the microfeatures. This prolate shape drives growth in the direction of the major axis of the ellipsoid growth cone which is in line with the ridges and grooves. The neurite shaft then orients in the same trajectory since the growth cone is uniformly driving growth in that direction.

It is evident that topographical features studied here change rDRGN growth cone behavior and that the growth cones have differential responses across topographical feature amplitudes, consistent with other studies [62, 63]. However, it is important to note that when imaged live the neurite shafts appear tremendously dynamic and reorient around the turns as the growth cone traverses the environment (supplementary video 2). The neurite shafts reorienting their position may obscure the inferences of our work since how a shaft is oriented at a given timepoint may not be representative of the path a growth cone took to create that neurite. Importantly, the conclusion that microfeature geometry determines neurite turning remains sound since even with the chaotic reorientation occurring, we still see clear and significant differences in our comparisons of interest (figures 2, 3 and supplementary figure 3). That being said, the reorientation of the neurite shafts is an important finding that motivates a reinterpretation of prior studies using fixed neurons to study pathfinding in response to various cues [17, 34]. Our data suggest that the growth cone may be consistently navigating a microfeature, but the shaft may reorient bringing it out of the microfeature. Therefore, tracing fixed neurites may not represent the true trajectory that the growth cone traversed in its elongation.

### 4.2. Neurite branching occurs when turning decision is uncertain

Beyond influencing neurite pathfinding, we also evaluated the relationship between the tendency of a neurite to branch and the ability of neurites to navigate a turn on these micropatterned surfaces [31, 64, 65]. We observed that branching occurred most often when the probability of neurites successfully making a given turn was essentially a tossup, near 50% (figure 7). When interpreted in combination with the live imaging data, these data suggest that the dynamic growth cones initiate branching at decision points on the micropatterned substrate. When the two paths offer equal resistance, i.e. making a turn or exiting out of the microfeature, both neurite branches are more likely to persist [66]. By contrast, on micropatterns with stronger guidance cues and greater certainty of growth behavior, the neurite either never initiates a branch, e.g. the growth cone remains in the microfeature for a gradual turn in a high amplitude feature, or ends up retracting the branch in the less favorable path, e.g. growth cone easily hops out a shallow microfeature at a sharp turn.

Though our work does not directly address the causes of branching, it complements other work that informs the process. First, others have indicated that mechanical force plays a significant role in neurite branching, in that neurons retract a growth cone branch if sufficient mechanical stress is applied [67]. In this context, mechanical stress on the growth cone from either the topographical microfeature ridge or turn likely initiates growth cone branching or dictates which arbor retracts. Additionally, topography has been implicated in branching frequency where topographical cues were presented to promote neurite branching [68]. The ability of specific cues to promote branching is consistent with the findings here that branching does not occur randomly, but can be stimulated to an extent by precise cues presenting a challenge with two equally likely directions of growth simultaneously. Overall, this angled microfeature system, or similarly constructed cues, offer a useful model to study neurite branching decisions.

### 4.3. Limitations of current work and future directions

This work is one of the first to use a precisely engineered topographically patterned substrate to systematically study neurite turning to angled and tunable microfeatures (figure 1), thus there are a few limitations of this initial work. First, neurons used in this study are derived from primary cell cultures, and cells on the micropatterned substrate are a heterogeneous mixture of the various cell types present in the ganglia. Therefore, the topographical growth cues inherent to the substrate are not the only guidance cues the neurites encounter in this system, as the glial cells may affect neurite guidance as well [54]. This tradeoff is necessary in order to study the pathfinding behavior of primary neurons with their full expression of receptors and channels. Second, all data are from neurons derived from neonatal animals. Neonatal SGNs are more dynamic compared to adult SGNs, the target cells of patients receiving CIs. This shortcoming is necessary as the yield and growth of SGNs from adult mice is limited. Likewise, growth cone size and dynamics are enhanced in neonatal DRGNs. Thus, using neonatal DRGNs for the live imaging and growth cone studies is necessary. That being said, adult SGNs and DRGNs have been shown to respond to topographical growth cues as well so the results are expected to translate [43]. A third potential shortcoming of the experimental system is the proximity of the angled turn cues to one another. While our system does not have simple, repeating cues arranged directly next to each other as in many other studies [30, 34], the cues are near each other and could impact behavior of the neurites. Importantly, our findings suggest that this impact is likely minimal. This is due to two observations. First, we use a variety of different observations to draw our conclusions, so it is unlikely that the neighboring channels influence the separate observations in the same manner across all experiments. In particular, these include observations of real time, local interactions of the growth cone with the channel ridge (figure 4), which should be unaffected by adjacent channels. Another limitation of this work was the duration of real time studies. While the real time observations were only for 1.5 h, they were of adequate duration for the stated goals. In particular, we sought to observe the behavior differences in the growth cones in real time, so neurons were imaged continuously and robust differences between moderate feature geometries were observed. Future work would explore methods to increase the duration of imaging including more resilient model neuronal culture systems, which approximate primary cells [69, 70] or non-continuous imaging to minimize high intensity light exposure. Lastly, this work did not assess neuronal function after the neurites navigated these biophysically patterned turn challenges. Follow up work using this system ought to further assess neuron functionality by performing similar work as others have done with *in vitro* studies by guiding neurite growth toward an electrode array and assessing the excitability and functionality of the neurite after being guided around various angled turns [71, 72].

To conclude, it is important to discuss how these findings inform the next steps for the field such as translating these findings to *in vivo* applications as well as expanding the understanding of fundamental mechanisms underlying neurite pathfinding. The findings from this work demonstrate fundamental principles that will be essential for translational endeavors *in vivo,* as the microfeatures in our work have sloped edges, so when growth cones grow within a microfeature, growth mechanics will mimic closed tubes with similar curvature [19]. A first finding that will inform *in vivo* application is that based on the decreased ability of neurites to pathfind to sharper angle cues, turns should be engineered to be gradual and to minimize zig-zagging as neurite shafts appear to resist this reorientation. For example, this may favor a lateral wall placed CI electrode where the neurite would need a gradual turn from the organ of Corti. Second, since the growth cones change their morphology and migration behavior to drive pathfinding in response to these microfeatures, translational biomaterials may be better suited to have aligned pores to guide growth. This paradigm has been proposed to be advantageous for neural conduits when compared to a biomaterial with a random meshwork [36, 37]. Lastly, minimizing neurite branching at the electrode interface will be necessary to optimize signal resolution in next generation CI. Our work suggests that neurite branching occurs as a function of neurite growth uncertainty when presented with multiple paths of growth. Therefore, in future material designs, instructive growth guides should be of sufficient strength to minimize exuberant branching. This may be challenging to accomplish since branching may occur at positions with unclear cues, such as when entering and exiting the designed biomaterial. Thus, as with pathfinding, neurite branching must also be considered, and minimized, for optimal recapitulation of the architecture and function of native neural system where growth is being induced.

## 5. Conclusions

Understanding the ability of neurites to sense and turn in response to topographical cues is critical for informing the future design of neuroprostheses, such as CIs, neural conduits, and mechanisms for guiding neurite outgrowth *in vivo*. Specifically, determining the limits and characteristics of a regenerating neurite to be guided and turn is essential. To investigate these important elements of neural regeneration, we used the precision of photopolymerization to engineer a novel topographical substrate with microfeature turns of various geometries. This substrate enabled our assessment of neurite turning in response to a range of topographical cues. Notably, our observations revealed that the geometric characteristics of the angled microfeatures significantly influenced the ability of sensory neurons to track the features, with deeper features and gradual turns facilitating better navigation. However, the intricate relationship between pathfinding fidelity and feature geometry remains complex. Despite growth cones driving this pathfinding through altered morphology and behavior, their inherently random and dynamic nature occasionally leads to deviations from the strongest growth cues studied here. Additionally, neurite branching is related to turning likelihood and is most likely to occur when turning decision is most uncertain. Lastly, neurites continue to be dynamic as the growth cones navigate within microfeatures. Occasionally neurite shafts reoriented themselves, leaving the features and stretching across the turns. This implies that tracing fixed neurites may not represent the true trajectory that the growth cone traversed during elongation. In summary, the topographically micropatterned multi-angle substrate has proven to be an effective and versatile system for comprehensively assessing neurite turning and pathfinding in response to topographical cues. The fundamental principles of neurite pathfinding discovered in this study are essential for informing the design of translational systems aimed at guiding neurite growth *in vivo.*

## Data Availability

All data that support the findings of this study are included within the article (and any supplementary files).
